# Reference intervals for 26 common biochemical analytes in term neonates in Jilin Province, China

**DOI:** 10.1186/s12887-021-02565-8

**Published:** 2021-03-31

**Authors:** Kaijin Wang, Xuetong Zhu, Qi Zhou, Jiancheng Xu

**Affiliations:** 1 Department of Laboratory Medicine, First Hospital of Jilin University, Changchun, China; 2 Department of Pediatrics, First Hospital of Jilin University, Changchun, China

**Keywords:** Biochemical analytes, Neonates, Reference intervals, Enzymes, Lipids

## Abstract

**Background:**

Biochemical analytes provide information for neonatal disease management and therapy, and population-based reference intervals (RIs) are essential to accurately interpret laboratory test results. This study aimed to establish local RIs for biochemical assays in term neonates.

**Methods:**

A total of 195 healthy term neonates from birth to 3rd day were recruited as reference individuals prospectively. Analytes of 26 common biochemistries were measured using the VITROS 5600 Integrated System. The 3-level nested ANOVA was performed to assess the need for partitioning RIs of each analyte, and RIs were derived by a nonparametric method or robust method. Multiple regression analysis was used to evaluate specific correlations between the analytes and individual characteristics including age, gender, gestational age, birthweight and delivery mode.

**Results:**

There were no between-sex differences in all analytes, whereas there were significant between-day-age differences in 6 analytes. Small between-delivery-mode differences were observed in the results for potassium, phosphorus, and urea. The major related factor of most analytes was postnatal age. During the first 3 days, values of iron, lipids and lipoproteins increased; creatinine, urea, uric acid, creatine kinase and lactate dehydrogenase decreased; other analytes showed slight changes or relatively stable trends. Reference limits of some analytes, particularly lactate dehydrogenase and alkaline phosphatase, were significantly different from adult and pediatric groups.

**Conclusions:**

RIs of 26 common biochemical analytes are established for term neonates aged 0 to 3 days in northeast China. Additionally, it is suggested that age-related changes should be valued in the clinical decision-making process for newborns.

**Supplementary Information:**

The online version contains supplementary material available at 10.1186/s12887-021-02565-8.

## Introduction

Reference intervals (RIs) are determined from individuals in healthy status, serving as tools for interpreting laboratory results. Despite the well standardized measurements of major laboratory tests, it is difficult to establish harmonized and uniform RIs on a global scale. Previous research has found that the differences in RIs of analytes are due to their complex patterns of biological sources of variations, such as region, race, diet, and other individual characteristics [1, 2]. Data from several studies suggest that age- and sex- specific RIs are more reliable to provide clinical decision support in the pediatric population [3–5]. Moreover, differences in analytical systems and statistical procedures may result in the differences between RIs reported in different studies [6–9]. Every aspect should be considered during the determination of accurate RIs.

Recently, there has been renewed interest in neonatal RIs with increased attention to neonatal health screening and management [10, 11]. Anthropometry and laboratory data are useful for neonatal growth and nutrition assessment [12]. The common biochemical analytes are related to measuring the functional maturity of organ systems, monitoring the changes of electrolyte and acid-base status, and evaluating the ability of material absorption and metabolism. Hence they can not only help diagnose relevant diseases but also engage in pharmacokinetic research. The Ministry of Health P. R. China has published several industry standards which provide RIs for common clinical biochemistry and immunology tests in adults, while data about newborns are not available. A few recent studies have established neonatal RIs for biochemistry analytes based on the local population, preterm and low-birth-weight infants included [11, 13–15]. However, most clinical laboratories in China adopt RIs from textbooks, reagent inserts, or adult reference standards. These data might be obsolete, lack of any traceability, or established on mismatched population in race or age, and then would be misleading to interpretate laboratory results. Thus, this study aimed to investigate the RIs for newborns during the first days in Jilin Province, China.

The Clinical and Laboratory Standards Institute (CLSI) recommends a priori method to determine RIs, namely selecting healthy individuals defined by specific reference criteria as reference populations in advance [16]. Another commonly used method, posteriori selection process, is to establish RIs using a large clinical database. There is difficulty in retrospectively establishing RIs in newborns for lack of a precise definition. The criteria may differ between different analytes, and neonates are prone to complicated pathological conditions during hospitalization. However, recruitment of healthy infants is also difficult because of ethical constraints for collecting unnecessary blood samples. We conducted this research with written informed consent from parents, and the specimen collection procedure was evaluated by qualified pediatricians to ensure rationality and applicability in local medical practice.

It is now widely acknowledged that term infants are different from preterm infants in the measured values of most biochemistry analytes [17–19]. Meanwhile, the two groups of infants have quite different causes of hospitalization. The premature are more susceptible to severe diseases for their immature organ function; whereas the common reasons for term infants to be hospitalized are birth injury, feeding intolerance, jaundice, sepsis and other medical complications. With this background, doctors should choose rational sets of tests and assess the results according to different criteria. In addition, most studies focus on establishing RIs and analyzing the correlations between individual parameters and analytes in newborns, who are born preterm or admitted to the neonatal intensive care unit. However, detailed data about term infants is limited.

Therefore, this prospective study aimed to determine RIs of 26 common biochemical analytes for term newborns during the first 3 days, and investigate the associations between the analytes and neonatal characteristics.

## Materials and methods

### Ethics

This study was approved by the Ethics Committee of the First Hospital of Jilin University, and written informed consent was obtained from all the babies’ parents.

### Study participants

Recruitment of subjects was from the obstetric unit of the First Hospital of Jilin University between July 2018 and October 2018, with 195 healthy term neonates enrolled prospectively. Neonatal assessment was performed by neonatal physicians, and maternal medical records were also reviewed. Eligibility criteria included gestational age between 37 and 42 weeks, birthweight ≥2500 g and < 4000 g, Apgar score at 1 and 5 min ≥7, and the newborn was delivered by normal delivery or elective caesarean section. Infants were excluded from this study if they had congenital infections, chromosomal abnormalities or severe neonatal diseases diagnosed in the perinatal period, as well as infants of mothers suffering from hypertension, thyroid dysfunction or other severe illnesses. All newborns were routinely vaccinated with hepatitis B and Bacillus Calmette-Guérin (BCG) after birth, and given vitamin K1 1 mg intramuscularly.

### Sample collection and laboratory procedure

Serum samples of newborns were taken within the first 3 days of life after obtaining informed consent from parents. A 4 ml sample of blood was collected into serum separation tubes in the morning from radial artery using a disposable butterfly needle. The samples were transported to the laboratory and centrifuged at 1200×g for 10 min within 1 h after collection. Specimens that were icteric, hemolyzed, or lipemic were removed prior to analysis, and then the activity or concentration of 26 biochemical analytes were determined on the VITROS 5600 Integrated System (Ortho Clinical Diagnostics/ Johnson & Johnson, Raritan, NJ, USA) within 2 h after separation. The analytes were as follows: total carbon dioxide (CO_2_), chloride (Cl), potassium (K), sodium (Na), calcium (Ca), iron (Fe), magnesium (Mg), phosphorus (P), creatinine (Cr), urea (BUN), uric acid (UA), alanine aminotransferase (ALT), alkaline phosphatase (ALP), aspartate aminotransferase (AST), cholinesterase (CHE), creatine kinase (CK), creatine kinase MB form (CK-MB), gamma glutamyl transferase (GGT), lactate dehydrogenase (LDH), albumin (Alb), total protein (TP), transferrin iron-binding capacity (TIBC), cholesterol (TCHO), high-density lipoprotein-cholesterol (HDL-C) and triglycerides (TG). The low-density lipoprotein-cholesterol (LDL-C) result was calculated with the Friedewald equation for SI units [LDL-C = TC − HDL-C−(TG/2.2)]. All reagents and quality control products for the assays were purchased from Ortho Clinical Diagnostics/ Johnson & Johnson company. Qualified laboratory proficiency and staff training were described in the research conducted in the same period [6, 8]. Analytical procedures were performed according to the manufacturer’s instructions and laboratory protocols, including regular maintenance, function checks, calibration, and internal quality control. Details of analytical methods and performance were listed in Supplemental Table 1.

### Statistical analysis

The study population was divided into subgroups according to delivery mode, sex and day age. Histogram and Shapiro-Wilk test were performed to detect data distribution before statistical analysis, and non-normal data was logarithmically transformed. Demographic data were expressed as mean ± standard deviation or number when appropriate. Mann–Whitney test or Wilcoxon rank sum test was used to compare numerical variables; Chi-square test was used for categorical variables.

For all the biochemical analytes, outliers were eliminated using the Dixon D/R ratio rule first, and data were expressed as median and interquartile range. Percentile curves were generated for all analytes using the vector generalized linear and additive models (vgam package) [20, 21] with the LMS Quantile Regression function. Three-level nested ANOVA was applied to evaluate sources of variation of RIs [22]. The magnitude of between-subgroup differences partitioned by the factors was computed as the standard deviation (SD) ratio (SDR), which corresponded to the SD from between-subgroup variation divided by SD between individuals. The variations of between-delivery-mode, between-sex and between-day-age components were expressed as SDR-dm, SDR-sex and SDR-age respectively. A ratio greater than 0.40 was considered as the criterion to partition RIs, in order to reduce bias caused by a narrow measure of between-individual variation [1, 23]. According to CLSI EP28-A3c guidelines [16], the lower limits (LL) and upper limits (UL), referring to 2.5th percentiles and 97.5th percentiles, were derived by the nonparametric method or robust method after deciding the partition. The 90% confidence intervals (CIs) for reference limits were estimated on the bootstrap method through iterative resampling 1000 times.

Multiple linear regression was used to investigate the associations between the analytes and individual characteristics, including GA, birthweight, delivery mode, sex and postnatal age. The significance of each variable was evaluated as standard partial regression coefficient (r_p_), which corresponded to partial correlation coefficient and takes value between − 1.0 and 1.0. Based on an objective classification system in previous studies [24, 25], the practical correlation was characterized as slight for 0.20 ≤ |r_p_| < 0.30, moderate for 0.30 ≤ |r_p_| < 0.50 and strong for 0.50 ≥ |r_p_|. All statistical analyses were performed using the *R* statistic software (version 3.6.0) and MedCalc Software (version 15.2). A bilateral *P*-value < 0.05 indicated statistical significance.

## Results

### Characteristics of study participants

A total of 195 term infants with postnatal age of 1–3 days fulfilled the inclusion criteria, and demographic information was shown in Table 1. Overall, both sexes were approximately equally represented in the study population, whereas the percentage of babies by caesarean section and on the second day was relatively large, accounting for 86.7 and 61.5% respectively. In brief, sex, birthweight, gestational age, maternal age and delivery mode did not show significant differences among the day-age groups.

**Table 1:** Participant demographics

Characteristics	Total	Day 1	Day 2	Day 3	***P*** value
	*n* = 195	*n* = 21	*n* = 120	*n* = 54	
Gestational age (weeks)	38.7 ± 1.1	39.0 ± 0.86	38.6 ± 1.1	38.7 ± 1.1	0.17
Birthweight (kg)	3.37 ± 0.43	3.58 ± 0.44	3.33 ± 0.45	3.37 ± 0.39	0.13
Boys/girls	104/91	12/9	62/58	30/24	0.83
Vaginal delivery/caesarean section	26/169	5/16	14/106	7/47	0.31

Data represented as mean ± standard deviation or number when appropriate

### Trends and RI establishment for common biochemical analytes

**Fig. 1:**
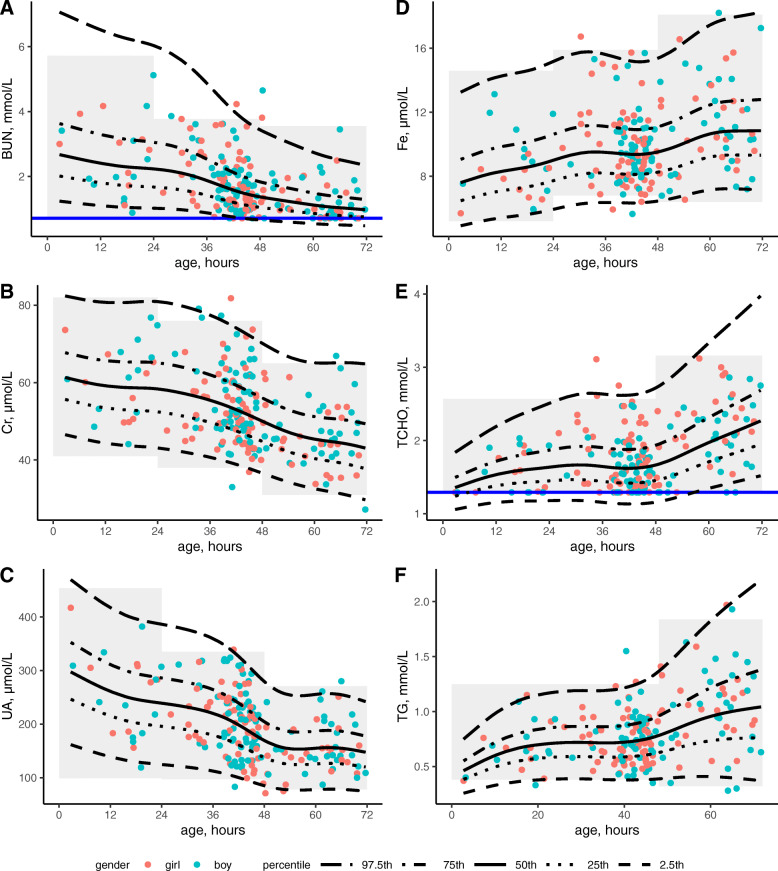
Percentile charts and partitioned RIs for BUN, Cr, UA, Fe, TCHO and TG. The RIs are indicated in grey area. Horizontal blue lines shown in the graphs for BUN and TCHO represent lowest detection limit

The division of RIs was codetermined by the scatter plot and 3-level nested ANOVA. Twenty-six common biochemical analytes had been measured during the first 3 days of life, and variation tendency depicted with the smoothed percentile curves for all analytes. Figure 1 and supplement Figures presented some interesting patterns, 1) BUN, Cr and UA significantly decreased over time, whereas Fe increased gradually within the 3 days; 2) there was an overall slight decrease in AST, CK, GGT, LDH, CK-MB, CO_2_ and TIBC, while ALP showed a minor increase; 3) during the first day of life, Alb, TP, CHE and K decreased slightly, whereas Cl and Na increased slightly, and then they held steady in the following days; 4) TCHO, HDL-C, LDL-C and TG seemed to be steady during the first 2 days and began to rise on the 3rd day; 5) Ca appeared to decline within 2 days and then leveled off, yet Mg and P represented an opposite trend; 6) ALT remained steady all the time.

**Fig. 2:**
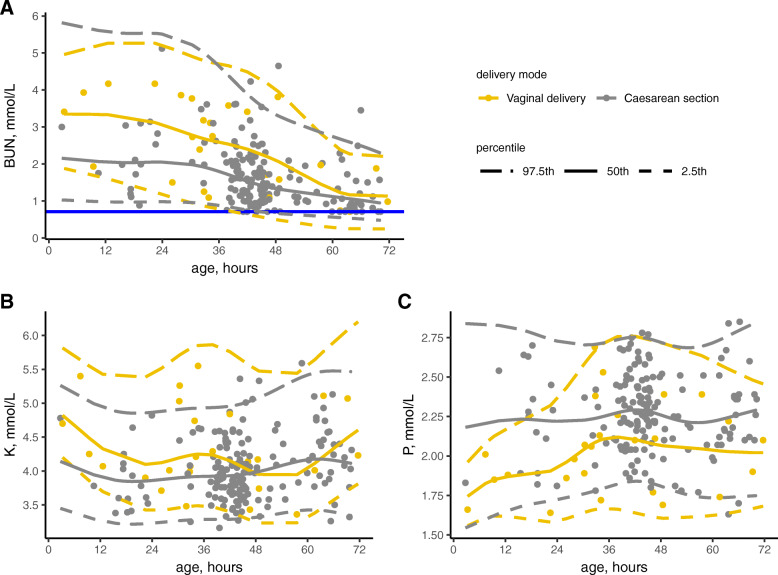
Percentile charts for K, P and BUN between subgroups in different delivery modes. Horizontal blue lines shown in the graphs for BUN represent lowest detection limit

By adopting 0.40 as a significant effect size for SDR, the result of 3-level nested ANOVA showed that none of the analytes was affected by sex. The SDR values for delivery mode (SDR-dm) were higher than 0.40 for K, P and BUN. As shown in Fig. 2, the upper limit (UL) and lower limit (LL) of subgroups divided by delivery mode were similar for BUN; instead, the BUN decreased significantly in a time-dependent manner. In this case, the RI of BUN was not partitioned by the mode of delivery. When the RIs of K and P were analyzed for vaginal births (VD) and cesarean-section (C/S) groups respectively, the UL of VD group (RI: 3.2–5.9 mmol/L) for K was higher than that of C/S group (RI: 3.3–5.4 mmol/L); the LL of VD group (RI: 1.56–2.79 mmol/L) for P was lower than that of C/S group (RI: 1.78–2.77 mmol/L). A small numerical distance was acceptable according to the clinician’s recommendation and the level of clinical decision limit for both, the combined RIs of K and P were calculated to be 3.3–5.4 mmol/L and 1.70–2.75 mmol/L.

From Table 2, the differences among day-age groups expressed as SDR-age were highlighted for 6 analytes. The medians and ULs of Fe, TCHO and TG gradually increased with age, however the changes of LLs were not very noticeable; the RIs for renal function tests (BUN, Cr and UA) went an apparent decline (Fig. 1). Age-specific RI for each analyte was summarized in Table 3.

**Table 2:** Result of 3-level Nested ANOVA and summary of statistical description for 26 biochemical analytes according to day age

Analytes	3-level Nested ANOVA			Day 1		Day 2		Day 3		Total	
	SDR-dm	SDR-sex	SDR-age	median	IQR	median	IQR	median	IQR	median	IQR
CO_2_, mmol/L	0.221	0.000	0.100	24	22-25	21	20-23	21	19-25	22	20-24
Cl, mmol/L	0.222	0.000	0.319	101	95-104	103	100-104	102	97-105	102	98-104
K, mmol/L	0.431	0.000	0.231	3.9	3.6-4.3	4.0	3.7-4.3	4.2	3.8-4.5	4.0	3.7-4.4
Na, mmol/L	0.000	0.000	0.021	135	133-136	135	133-137	135	133-136	135	133-137
Ca, mmol/L	0.285	0.000	0.000	1.97	1.88-2.01	1.92	1.83-2.03	1.96	1.81-2.04	1.95	1.83-2.03
Mg, mmol/L	0.264	0.000	0.323	0.77	0.76-0.80	0.80	0.76-0.84	0.80	0.78-0.87	0.80	0.76-0.84
P, mmol/L	0.673	0.139	0.091	2.11	1.85-2.36	2.28	2.10-2.43	2.18	2.07-2.39	2.25	2.07-2.42
Fe, μmol/L	0.151	0.000	0.411	8.4	7.1-9.4	9.4	8.3-11.0	10.6	8.7-12.8	9.6	8.3-11.3
TIBC, μmol/L	0.278	0.000	0.000	42.0	40.0-44.4	40.4	37.8-43.0	39.9	37.2-43.7	40.5	37.7-43.3
BUN, mmol/L	0.580	0.000	0.519	2.53	1.75-3.14	1.65	1.14-2.25	1.08	0.78-1.63	1.57	1.05-2.15
Cr, μmol/L	0.121	0.000	0.670	61	52-67	52	48-60	46	42-52	51	45-60
UA, μmol/L	0.000	0.000	0.675	278	183-308	209	164-244	146	130-189	186	145-241
ALT, U/L	0.164	0.000	0.000	30	27-32	30	22-35	31	25-34	30	24-34
ALP, U/L	0.096	0.228	0.000	143	129-155	150	123-167	156	141-172	151	127-168
AST, U/L	0.000	0.272	0.180	56	52-83	52	44-63	52	42-63	53	44-63
CHE, U/L	0.325	0.000	0.167	4326	4060-4983	4266	3871-4696	4272	4069-4458	4279	3897-4655
GGT, U/L	0.000	0.000	0.000	133	91-169	129	86-169	113	88-142	122	86-169
CK, U/L	0.000	0.213	0.251	412	297-470	318	233-414	238	157-382	304	203-415
CK-MB, U/L	0.066	0.116	0.000	22	18-26	24	18-30	22	18-29	23	18-30
LDH, U/L	0.000	0.146	0.246	1247	972-1330	1149	1022-1270	1039	928-1230	1120	985-1285
Alb, g/L	0.263	0.215	0.116	33.7	30.8-36.3	32.7	30.8-34.3	32.3	30.3-34.7	32.7	30.7-34.7
TP, g/L	0.314	0.190	0.000	47.1	45.6-50.8	48.0	45.5-52.0	49.6	45.6-52.2	48.2	45.5-52.1
HDL-C, mmol/L	0.117	0.000	0.315	0.62	0.55-0.80	0.67	0.58-0.81	0.71	0.59-0.83	0.68	0.58-0.82
LDL-C, mmol/L	0.000	0.000	0.319	0.56	0.51-0.80	0.57	0.45-0.80	0.90	0.56-1.15	0.63	0.48-0.91
TCHO, mmol/L	0.125	0.000	0.618	1.57	1.29-1.85	1.60	1.38-1.92	2.08	1.65-2.39	1.73	1.39-2.05
TG, mmol/L	0.000	0.000	0.517	0.64	0.56-0.77	0.70	0.58-0.86	1.02	0.77-1.26	0.74	0.58-0.95

SDR ≥ 0.4 was used as a criterion for partition of reference values

*SDR* standard deviation ratio, *SDR-dm* SDR for between-delivery-mode differences, *SDR-sex* SDR for between-sex differences, *SDR-age* SDR for between-day-age differences, *IQR*, interquartile range

**Table 3:** Age-dependent reference intervals for 26 biochemical analytes in newborns

Analytes	Age	Lower Limit (90% CI)	Upper Limit (90% CI)
Chemistry			
CO_2_, mmol/L	1–3 d	16 (16–17)	28 (28–29)
Cl, mmol/L	1–3 d	92 (91–93)	108 (108–109)
K, mmol/L	1–3 d	3.3 (3.2–3.4)	5.4 (5.1–5.6)
Na, mmol/L	1–3 d	131 (130–131)	140 (140–141)
Ca, mmol/L	1–3 d	1.53 (1.42–1.61)	2.15 (2.12–2.16)
Mg, mmol/L	1–3 d	0.68 (0.65–0.70)	0.94 (0.91–0.96)
P, mmol/L	1–3 d	1.70 (1.64–1.78)	2.75 (2.68–2.84)
Fe, μmol/L	1 d	5.2 (4.6–5.9)	14.6 (12.1–17.2)
	2 d	6.8 (6.6–7.0)	15.9 (14.6–17.4)
	3 d	6.4 (5.9–7.1)	18.1 (16.3–19.8)
TIBC, μmol/L	1–3 d	32.9 (31.9–35.1)	52.4 (50.5–55.5)
BUN, mmol/L	1 d	0.71^a^	5.72 (4.58–6.87)
	2 d	0.71^a^	3.77 (3.58–4.23)
	3 d	0.71^a^	3.55 (2.56–5.04)
Cr, μmol/L	1 d	41 (35–47)	82 (76–87)
	2 d	38 (37–40)	76 (72–80)
	3 d	31 (29–34)	65 (60–69)
UA, μmol/L	1 d	99 (68–151)	454 (388–512)
	2 d	97 (87–110)	335 (318–351)
	3 d	78 (69–88)	271 (236–300)
Enzymes			
ALT, U/L	1–3 d	6^a^	44 (41–46)
ALP, U/L	1–3 d	91 (83–103)	229 (214–240)
AST, U/L	1–3 d	29 (29–33)	101 (92–115)
CHE, U/L	1–3 d	3299 (3272–3397)	6491 (5838–6736)
GGT, U/L	1–3 d	42 (39–54)	302 (280–316)
CK, U/L	1–3 d	95 (89–109)	715 (566–773)
CK-MB, U/L	1–3 d	12 (11–13)	40 (39–41)
LDH, U/L	1–3 d	651 (570–720)	1577 (1524–1620)
Proteins			
Alb, g/L	1–3 d	28 (27–28)	40 (39–43)
TP, g/L	1–3 d	41 (40–41)	59 (57–61)
Lipids/Lipoproteins			
HDL-C, mmol/L	1–3 d	0.43 (0.39–0.46)	1.13 (1.04–1.19)
LDL-C, mmol/L	1–3 d	0.20 (0.17–0.22)	1.48 (1.26–1.76)
TCHO, mmol/L	1–2 d	1.29^a^	2.57 (2.46–3.11)
	3 d	1.29^a^	3.16 (2.96–3.37)
TG, mmol/L	1–2 d	0.38 (0.33–0.43)	1.25 (1.12–1.55)
	3 d	0.32 (0.22–0.44)	1.84 (1.66–2.01)

^a^Lowest detection limit. Actual values could be lower

### MRA to evaluate the correlations between individual characteristics and biochemical analytes

Multiple linear regression analysis (MRA) in Table 4 showed consistent results with nested ANOVA analysis. For newborns, there was no actually significant association between sex and analyte concentrations during perinatal period. Instead, age was the main source of variation related to most analytes. In the regression models, some analytes showed slight associations with the delivery mode, birthweight and gestational age (|r_p_|<0.3). Infants born by vaginal delivery had a slightly high level of K and BUN and a slightly low level of P, compared to cesarean delivery. For BW-related changes, birthweight was positively correlated with P and TIBC and negatively correlated with ALT and ALP; for GA-related changes, GA showed positive correlations with ALT, CK and TP.

**Table 4:**
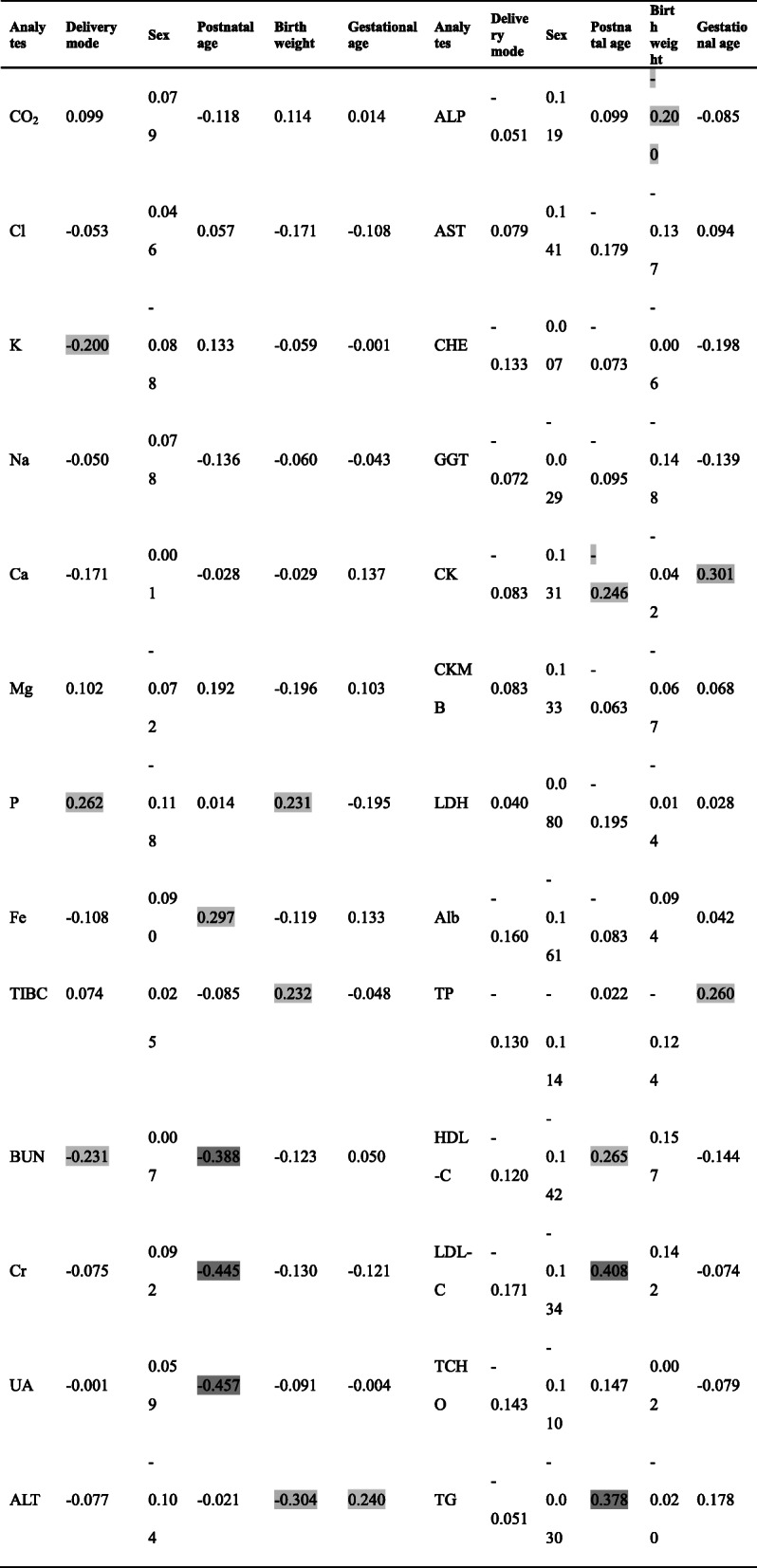
Multiple linear regression of individual characteristics on the biochemical analytes

Listed values are standardized partial regression coefficients (r_p_). |r_p_| values that exceed 0.2 was shown in three graded black background colors: light 0.2 ≤ |r_p_|<0.3, moderate 0.3 ≤ |r_p_| ≤ 0.5, dark |r_p_| ≥ 0.5 respectively

### RIs comparison to other studies

**Fig. 3:**
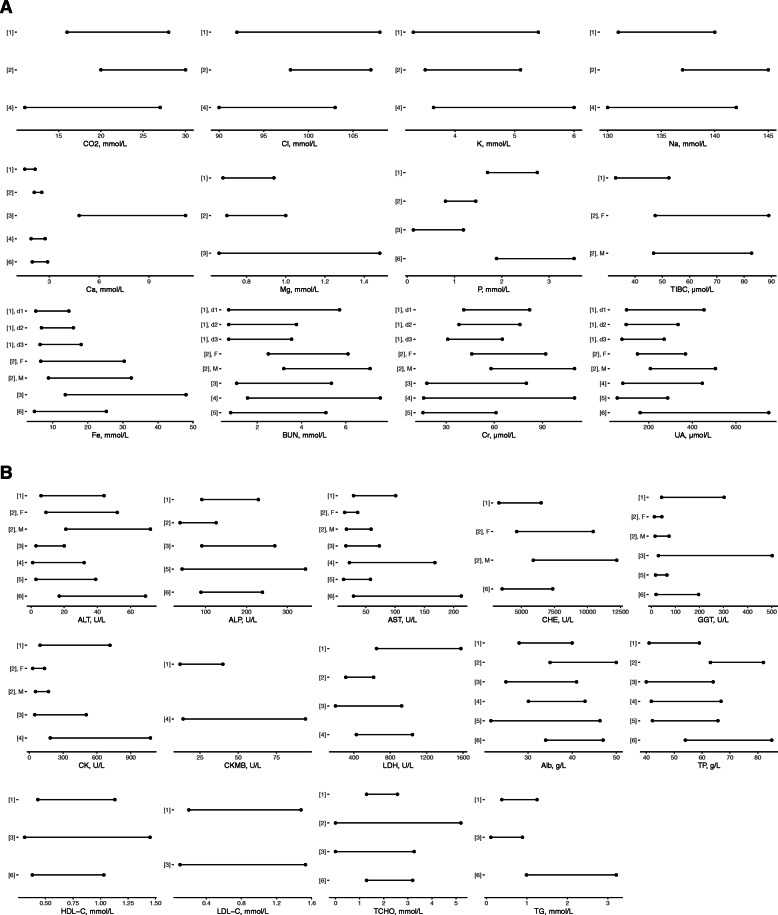
Biochemical reference intervals in comparison with other studies. [1] represents the current study, [2] represents kit insert (F for female, M for male), [3] represents Choi et al., [4] represents Zhao et al., [5] represents Liu et al., [6] represents Higgins et al

The RIs of the 26 biochemical analytes showed differences among this study, reagent inserts and other published data (see Fig. 3 and Supplemental Table 2). Comparing with the adult RIs provided by manufacturers, Cl, K, Na, and Mg did not show significant differences; lower ULs and LLs for CO_2_, Ca, Fe, TIBC, BUN, Cr, UA, ALT, CHE, Alb and TP were observed in healthy neonates, however higher values for P, ALP, AST, GGT, CK, LDH could be seen. In particular, the analysis of CK-MB, lipid or lipoprotein was complex in adults, therefore direct comparisons of the RIs were omitted.

In comparison with the RIs derived from other experimental designs, sex partitions were not emphasized in all the researches, but different age divisions and reference limits were able to be observed. These results showed that some differences in LDH were quite marked among the RIs listed for similar age groups. ULs of Ca, Mg, Fe, TCHO and HDL-C are significantly lower than those from the experiment based on the cord blood in Korean [13]. The RIs of most analytes are similar to those from the study in Lanzhou, China [15], however the RIs of liver enzymes except CHE showed great differences compared with those from Zhengzhou, China [26].

## Discussion

This study described the distribution and dynamic changes of biochemistry analytes from healthy term infants during the first 3 days of life in northeast China. There was heterogeneity in analytes with different biological sources of variations, however, day age showed a predominant correlation with the concentration for most analytes. Premature and term infants were prone to different diseases, and the demand for clinical evaluation and nursing intervention varied [27]. Therefore, the local RIs in term infants were derived for ensuring a high accuracy of clinical management.

### Age-dependent RIs for 26 common biochemical analytes

In reviewing the literature, gestational age and birthweight were regarded as key factors for establishing RIs for preterm infants, but little difference of the two factors would be found in healthy term newborns. Although the guidance documents for establishing RIs only set the lowest limit for the number of subgroup participants, there was no single, specific definition for age intervals. If the age intervals were too wide, the rapid changes in a short time might be otherwise overlooked. Therefore, this study statistically depended upon SDR-dm, SDR-sex and SDR-age to analyze differences between subgroups, and then judged whether it was appropriate for clinical practice to partition RIs according to delivery mode, sex and postnatal age. Regarding the test results of SDR-dm and SDR-sex, RIs of all items did not require specific partitions (SDR < 0.40) except for K, P and BUN. It was suggested that the combination of scatter plots and SDR values decide whether to partition RIs for test results compared across different groups. This helps avoid exaggerated SDR for the actual narrow range of RIs or masked differences caused by the interaction between the factors [2, 28]. In effect, no sex difference for all analytes was detected, which was consistent with data in different regions [5, 11, 13].

However, there were some considerable differences in postnatal age. This might be due to the rapid physiological adaptation to extrauterine life for newborns. A possible explanation for this might be that perinatal data was mainly affected by maternal and neonatal physiological factors during the first days of life. The concentration of each analyte reflected the balance between production, metabolism and clearance. First, some easy-to-understand mechanisms may cause short-term fluctuations of most analytes, such as proteins, electrolytes, lipids [29] and renal function markers [30], listed as follows: 1) the exchange of substances based on placental transfer between the fetus and mother ceased abruptly after delivery; 2) developmental and maturational changes occurred in the organs during the perinatal period, especially liver and kidney; 3) the intake of external nutrients directly and indirectly affected the concentration of metabolites. Furthermore, the stress response during labour can also have an impact. Enzyme levels evaluated in our study were consistent with Lackmann et al. who found that the cytoplasmic and mitochondrial enzymes presented similar activity curves, whereas the membrane-bound enzymes showed the opposite. The differences in enzymes were considered the result of minor cell damage caused by physiological hypoxia during labour [31]. However, the changes in our study may be not obvious enough because the release of enzymes was also affected by uterine contractions and physical stress through the birth canal, while the small number of natural delivery were included [32]. The regulation of substances by hormones was equally important after birth, which can be demonstrated by bone metabolism status in neonates. In addition to maternal vitamin D during pregnancy [33], serum Ca, Mg and P homeostasis was also regulated by foetal parathyroid hormone (PTH), calcitriol, calcitonin, calcium sensing receptor (CaSR) and fibroblast growth factor-23 (FGF-23), which affecting bone physiology, intestinal absorption and renal excretion [34].

### Other variables that related to RIs of biochemical analytes

Generally the main considerations for sources of variations of each test were sex, age and BMI in adults and children, besides races and regions. In the case of neonates, even more factors came into play, including maternal and infant health, delivery mode, gestational age and weight at birth. The comparison between the MLA model and the nested ANOVA method indicated that the results of delivery mode and sex were almost the same in present study, and postnatal age was the main influence on the concentration of most analytes. Birthweight and gestational age were widely used in evaluating neonatal clinical outcomes, while both had limited effects on a few items. A possible explanation for this might be that the premature or overdue babies and the low birthweight or fetal macrosmia were not included in this study.

In this study, there was poor negative correlation between birthweight and ALT and ALP, but positive correlation with P and TIBC although in term healthy newborns. Surprisingly, the small but consistent inverse associations of birthweight with ALT and ALP remained in late adolescent [35] and adult [36]. In fact, an elevated level of ALT within the normal range was proven to predict the hepatic steatosis of metabolic syndrome [36, 37]. It may be assumed that birthweight is a proxy for exposures such as intrauterine nutrition or genetic factors that directly affect the liver. In addition, the opposite correlation of birthweight with ALP and P may reflect the inverse association between birthweight and bone strength. For another, the observed increasing levels in CK, TP and ALT with GA could be attributed to the rate of the babies’ muscular mass, or to the development of their metabolism in advanced pregnancy [19, 38]. It is noteworthy that the expression of LC3-II and p62 in cord blood was associated with serum total protein in infants, perhaps suggesting that the autophagy reaction introduced by postnatal starvation played a crucial part in the maintenance of protein or amino acid metabolism during the perinatal period [39].

### Comparison to other studies

RIs in our study were compared with those of kit inserts and other studies, which covered different experimental types and designs, assay systems, characteristics of the studied population and specimen types. The manufacturer RIs were less reliable in the evaluation of clinical applicability before use in Chinese laboratories, because they were generally based on Caucasian people and lacked data on children. The RIs of Cl, K and Na were similar to those from reagent inserts and previous findings for children and adolescents [8]. Therefore, it seemed that the three electrolytes remained relatively stable in one’s life. The differences of RIs for Ca, Mg, P and lipids between this study and a study in Korea, to some extent, might be explained by the fact that umbilical cord contained maternal substances for fetal development. Compared to another two studies in other parts of China, there might be some factors that contributed to the differences between RIs, including actual differences between cities, laboratories, analytical systems and statistical calculation methods reported in these studies. Moreover, Liu et al. [26]used an indirect method for determination of RIs, which based on the stricter exclusion criteria and wider age intervals, and then little data about the rapid changes was presented during the first days.

As mentioned in the Caliper’s report about transferred RIs from Abbott to Ortho assays [40], Ca, CO_2_, Mg and LDH did not meet transference or verification criteria. Except these, the RIs of most corresponding analytes showed higher values, which might be attributed to racial and diet differences. This finding further support that laboratories should verify transferred RIs for local population and analytical platform—that covers as many partitioned RIs as possible—according to CLSI guidelines.

### Limitations

Unfortunately, several limitations existed. The current work was based on a small sampling of healthy newborns, and the distribution of data was unbalanced between subgroups. All the subjects contained a high number of neonates on the 2nd day and by caesarean section. Once the RI was partitioned, the small sample size would be hard to assure a highly accurate RI with narrow confidence intervals. Furthermore, this study just determined the RIs in newborns aged 0–3 days, and new data on infant period should be supplemented in the future. All the analytes should be evaluated on their validity of routine use in clinics and further investigated in preterm infants. The percentile charts provided might be integrated into the hospital and laboratory information system to implement new strategies for result display.

## Conclusion

This study used a priori method to investigate analyzer and reagent-appropriate RIs in term infants, with clear inclusion and exclusion criteria, standardized sample processing procedures and novel data analysis methods. Moreover, the research of establishing RIs on VITROS 5600 Integrated System was unique, and this study complemented those of earlier studies on children and adolescents. In summary, this study preliminarily established serum RIs for 26 common biochemical analytes in healthy term infants, investigated their dynamic changes during the first 3 days of life and evaluated the associations of individual characteristics with these analytes. The results of this study indicated that day-age-appropriate RIs should be considered for newborns in the field of perinatal-neonatal medicine. Continued efforts are needed to further assess the significance of these RIs in routine clinical work and disease diagnosis.

## Supplementary Information

**Additional file 1: Supplemental Table 1.** Analytical performance of chemistry assays on the Ortho VITROS 5600 Integrated System.

**Additional file 2.** Percentile charts and combined RIs for other biochemical analytes.

**Additional file 3: Supplemental Table 2.** Summary of reference intervals for 26 biochemistry analytes in comparison with other studies.

## Data Availability

The datasets used and/or analysed during the current study are available from the corresponding author on reasonable request.

## Abbreviations

CO_2_: Total carbon dioxide
Cl: Chloride
K: Potassium
Na: Sodium
Ca: Calcium
Fe: Iron
Mg: Magnesium
P: Phosphorus
Cr: Creatinine
BUN: Urea
UA: Uric acid
ALT: Alanine aminotransferase
ALP: Alkaline phosphatase
AST: Aspartate aminotransferase
CHE: Cholinesterase
CK: Creatine kinase
CK-MB: Creatine kinase MB form
GGT: Gamma glutamyl transferase
LDH: Lactate dehydrogenase
Alb: Albumin
TP: Total protein
TIBC: Transferrin iron-binding capacity
HDL-C: High-density lipoprotein-cholesterol
TCHO: Cholesterol
TG: Triglycerides
LDL-C: Low-density lipoprotein-cholesterol
LL: Lower limit
MRA: Multiple regression analysis
UL: Upper limit
SDR: Standard deviations ratio
r_p_: Partial correlation coefficient
RI: Reference interval
