# Supplementary material for: Reference intervals for 26 common biochemical analytes in term neonates in Jilin Province, China
Source: BMC Pediatr. 2021 Mar 31;21:156. doi: 10.1186/s12887-021-02565-8 (PMC8011145; doi:10.1186/s12887-021-02565-8)
Supplement: Supplementary file 1 — Additional file 1: Supplemental Table 1. Analytical performance of chemistry assays on the Ortho VITROS 5600 Integrated System. [file 12887_2021_2565_MOESM1_ESM.docx]

**Supplemental Table 1** Analytical performance of chemistry assays on the Ortho VITROS 5600 Integrated System

| **Analytes** | **Bias of accuracy, %** | **Precision** | | | | **Measuring Range** | **Accreditation criteria** | | **Analytical method** |
| --- | --- | --- | --- | --- | --- | --- | --- | --- | --- |
|  |  | Low level, % | | High level, % | |  |  |  |  |
|  |  | Within-day | Between-day | Within-day | Between-day |  | Bias* | Total error* |  |
| CO_2_, mmol/l | 0.98 | 1.28 | 3.43 | 1.38 | 3.26 | 5.0-40.0 | 3.0 | 10.0 | Enzymatic |
| Cl, mmol/l | 0.44 | 0.93 | 2.03 | 0.60 | 1.83 | 50.0–175.0 | 1.5 | 4.0 | Potentiometric |
| K, mmol/l | 0.40 | 1.14 | 1.65 | 0.98 | 1.52 | 1.00–14.00 | 2.0 | 6.0 | Potentiometric |
| Na, mmol/l | 0.38 | 0.77 | 1.42 | 0.60 | 1.46 | 75.0–250.0 | 1.5 | 4.0 | Potentiometric |
| Ca, mmol/l | -0.40 | 0.59 | 2.00 | 0.62 | 1.45 | 0.25–3.49 | 2.0 | 5.0 | Arsenazo III dye |
| Fe, μmol/L | 1.21 | 1.39 | 2.62 | 0.84 | 3.14 | 1.81–107.46 | 4.5 | 15.0 | Ferrozine colourimetic |
| Mg, mmol/l | 1.70 | 1.20 | 2.75 | 0.70 | 1.96 | 0.08-4.11 | 5.5 | 15 | Formazan dye |
| P, mmol/l | 1.71 | 1.15 | 1.59 | 0.94 | 1.51 | 0.16-4.20 | 3.0 | 10.0 | Phosphomolybdate/p-semidine HCI |
| Cr, μmol/L | 0.81 | 1.29 | 1.32 | 1.97 | 1.33 | 13–1238 | 5.5 | 12.0 | Enzymatic |
| BUN, mmol/l | 0.54 | 1.40 | 1.33 | 2.20 | 1.33 | 0.71–42.83 | 3.0 | 8.0 | Enzymatic |
| UA, μmol/L | -2.15 | 0.80 | 3.26 | 0.40 | 2.20 | 29.7–1011.2 | 4.5 | 12.0 | Enzymatic |
| ALT, U/L | -0.80 | 4.08 | 4.24 | 1.25 | 1.67 | 6–1000 | 6.0 | 16.0 | Enzymatic with pyridoxal-5-phopsphate |
| ALP, U/L | 1.74 | 3.68 | 2.71 | 3.96 | 2.98 | 20–1500 | 10.0 | 18.0 | Enzymatic |
| AST, U/L | 4.86 | 1.40 | 1.42 | 1.40 | 1.52 | 9.0-653 | 5.0 | 15.0 | Enzymatic with pyridoxal-5-phopsphate |
| CHE, U/L | 0.72 | 1.82 | 2.40 | 2.32 | 3.98 | 200–12500 | 5.0 | 15.0 | Enzymatic |
| CK, U/L | 4.85 | 2.15 | 4.20 | 1.79 | 3.27 | 21.5–1566.0 | 5.5 | 15.0 | Enzyme coupling method |
| CKMB, U/L | 5.51 | 5.18 | 5.20 | 4.30 | 4.31 | 2.7–300.0 | 7.0 | 22.0 | Immunoinhibition method |
| GGT, U/L | 0.19 | 3.22 | 4.98 | 2.42 | 1.80 | 10–1400 | 5.5 | 11.0 | Enzymatic |
| LDH, U/L | 1.91 | 2.30 | 2.33 | 1.85 | 1.90 | 100-2150 | 4.0 | 11.0 | Enzymatic (P→L) |
| Alb, g/L | -0.55 | 0.60 | 1.79 | 0.80 | 1.20 | 10.0–60.0 | 2.0 | 6.0 | Bromcresol green (BCG) dye |
| TP, g/L | -0.12 | 1.10 | 1.66 | 1.82 | 2.13 | 20.0–110.0 | 2.0 | 5.0 | Biurea method |
| TIBC, μmol/L | -0.47 | 0.60 | 1.79 | 0.98 | 2.20 | 15.2–116.4 | 3.0 | 8.0 | Ferrozine colourimetic |
| TCHO, mmol/l | 1.50 | 0.81 | 1.62 | 0.62 | 1.72 | 1.29-8.40 | 4.0 | 9.0 | Enzymatic |
| HDL-C, mmol/l | 2.60 | 2.00 | 3.00 | 1.49 | 2.23 | 0.13-2.84 | 4.0 | 9.0 | Enzymatic |
| TRIG, mmol/l | 2.00 | 0.84 | 1.68 | 0.90 | 1.32 | 0.11–5.93 | 5.0 | 14.0 | Enzymatic |

*Bias and total error(TE) criteria are from Analytical quality specification for routine analytes in clinical biochemistry published by the Ministry of Health of the People’s Republic of China; due to lack of criteria for Bu, CKMB, CO_2,_ CHE and TIBC, bias and TE were recommended by our own clinical laboratory. Coefficient of variation (CV) for within-day precision should be<1/4 TE, and CV for between-day precision should be<1/3 TE according to Guidance on the Application of Accreditation Criteria for the Medical Laboratory Quality and Competence in the Field of Clinical Chemistry published by the China National Accreditation Service for Conformity Assessment.
