# Supplementary material for: Reference intervals for 26 common biochemical analytes in term neonates in Jilin Province, China
Source: BMC Pediatr. 2021 Mar 31;21:156. doi: 10.1186/s12887-021-02565-8 (PMC8011145; doi:10.1186/s12887-021-02565-8)
Supplement: Supplementary file 2 — Additional file 2. Percentile charts and combined RIs for other biochemical analytes. [file 12887_2021_2565_MOESM2_ESM.pptx]

## Slide 1
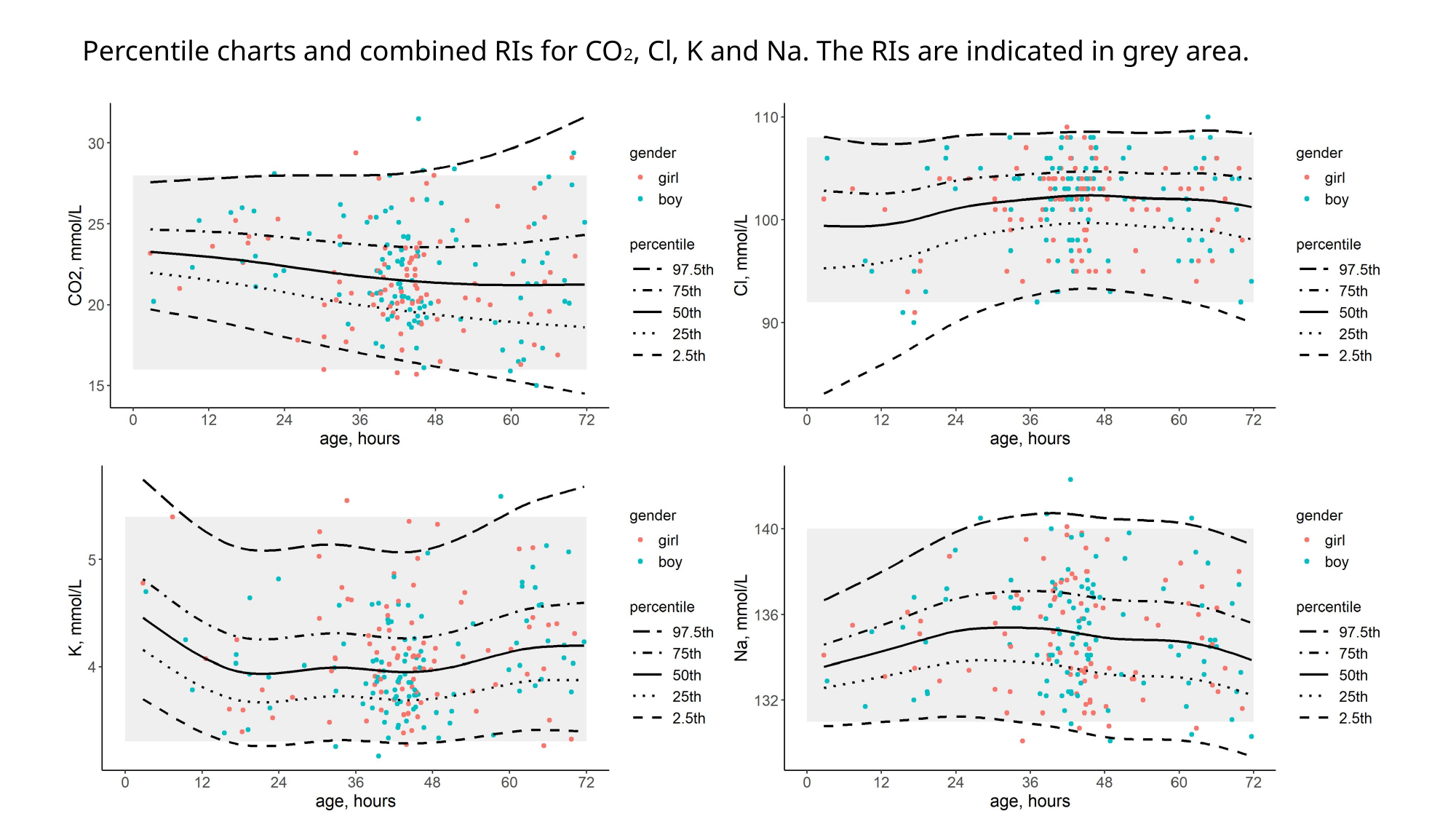

Percentile charts and combined RIs for CO2, Cl, K and Na. The RIs are indicated in grey area.

## Slide 2
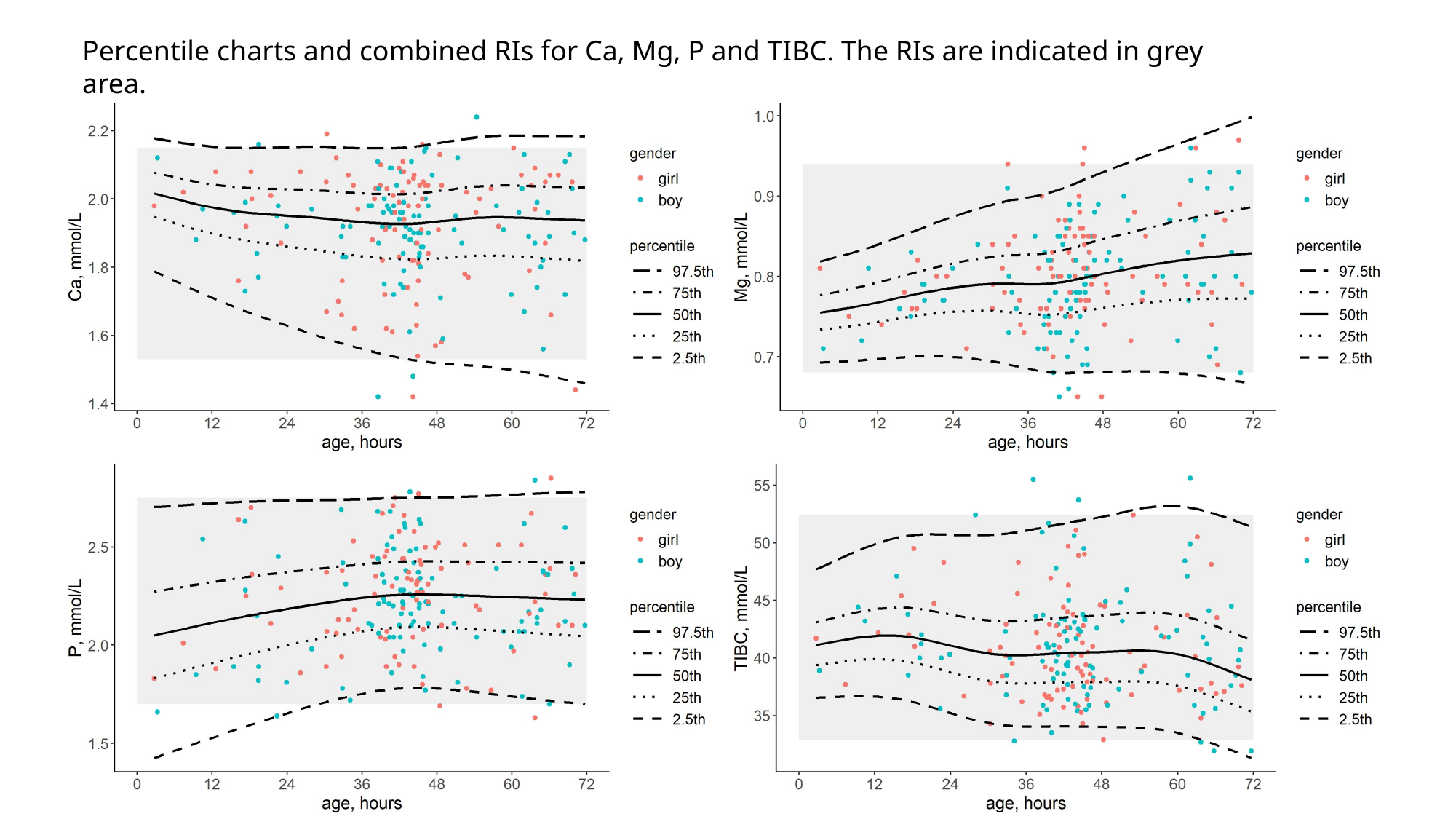

Percentile charts and combined RIs for Ca, Mg, P and TIBC. The RIs are indicated in grey area.

## Slide 3
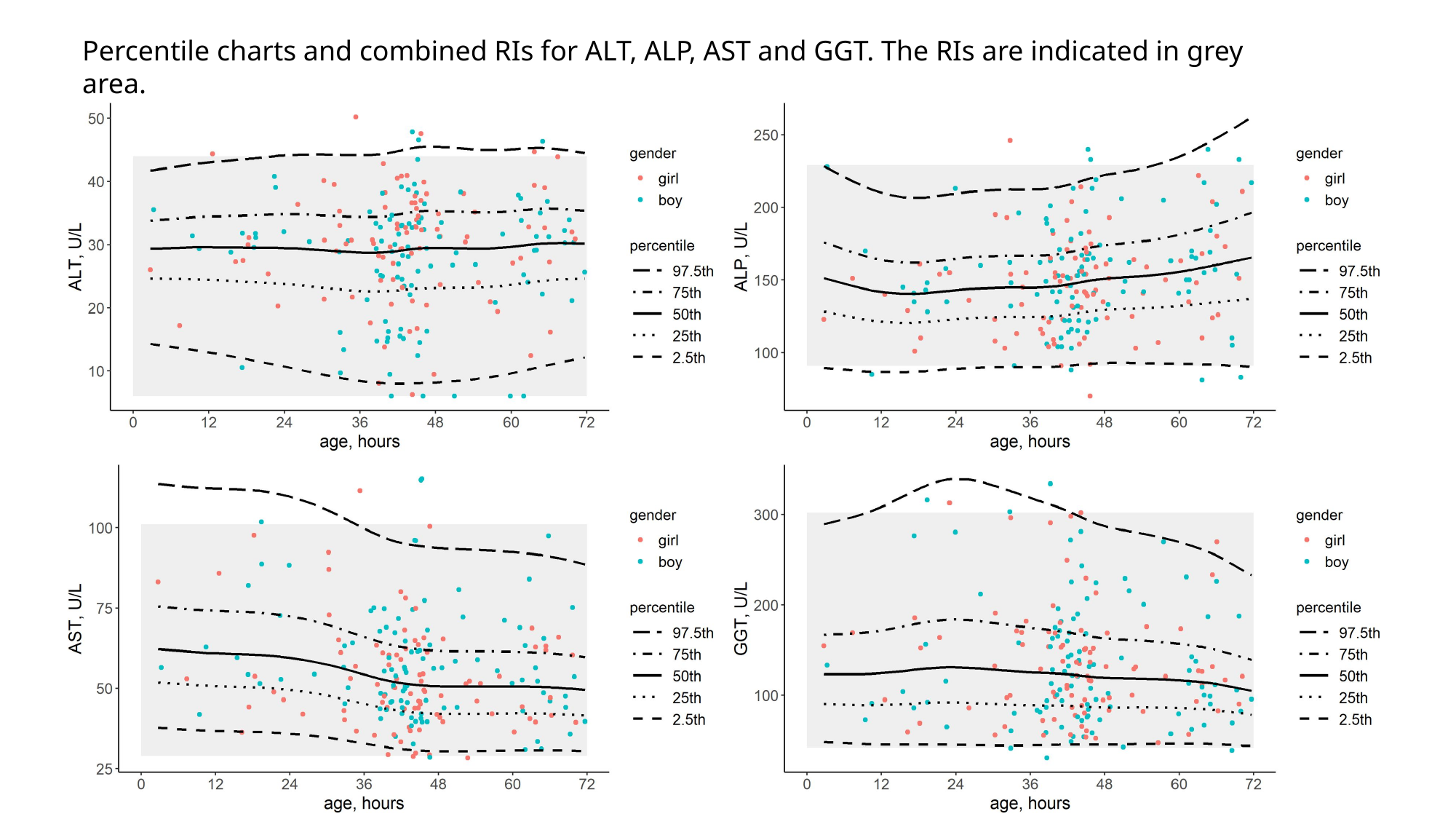

Percentile charts and combined RIs for ALT, ALP, AST and GGT. The RIs are indicated in grey area.

## Slide 4
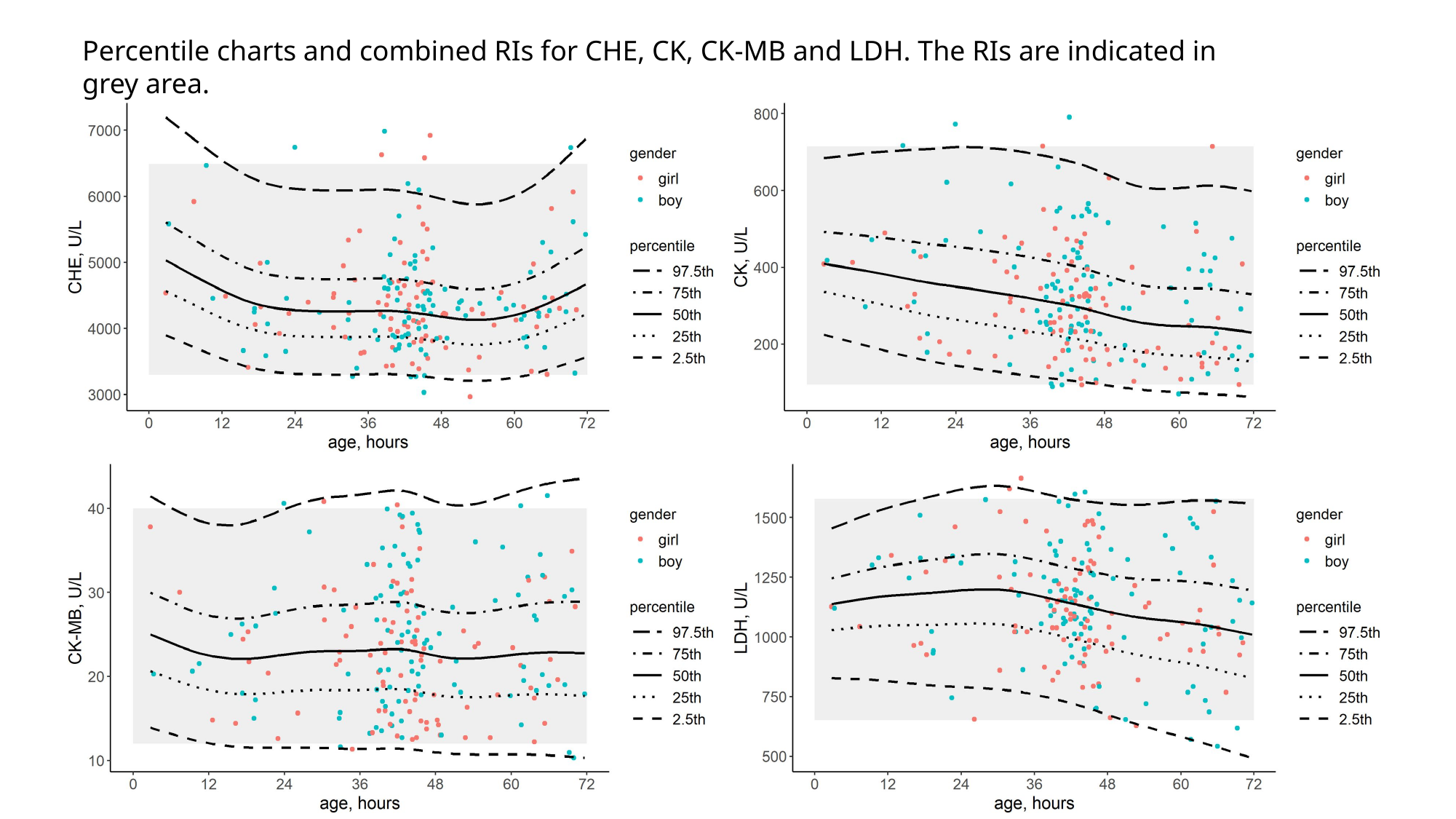

Percentile charts and combined RIs for CHE, CK, CK-MB and LDH. The RIs are indicated in grey area.

## Slide 5
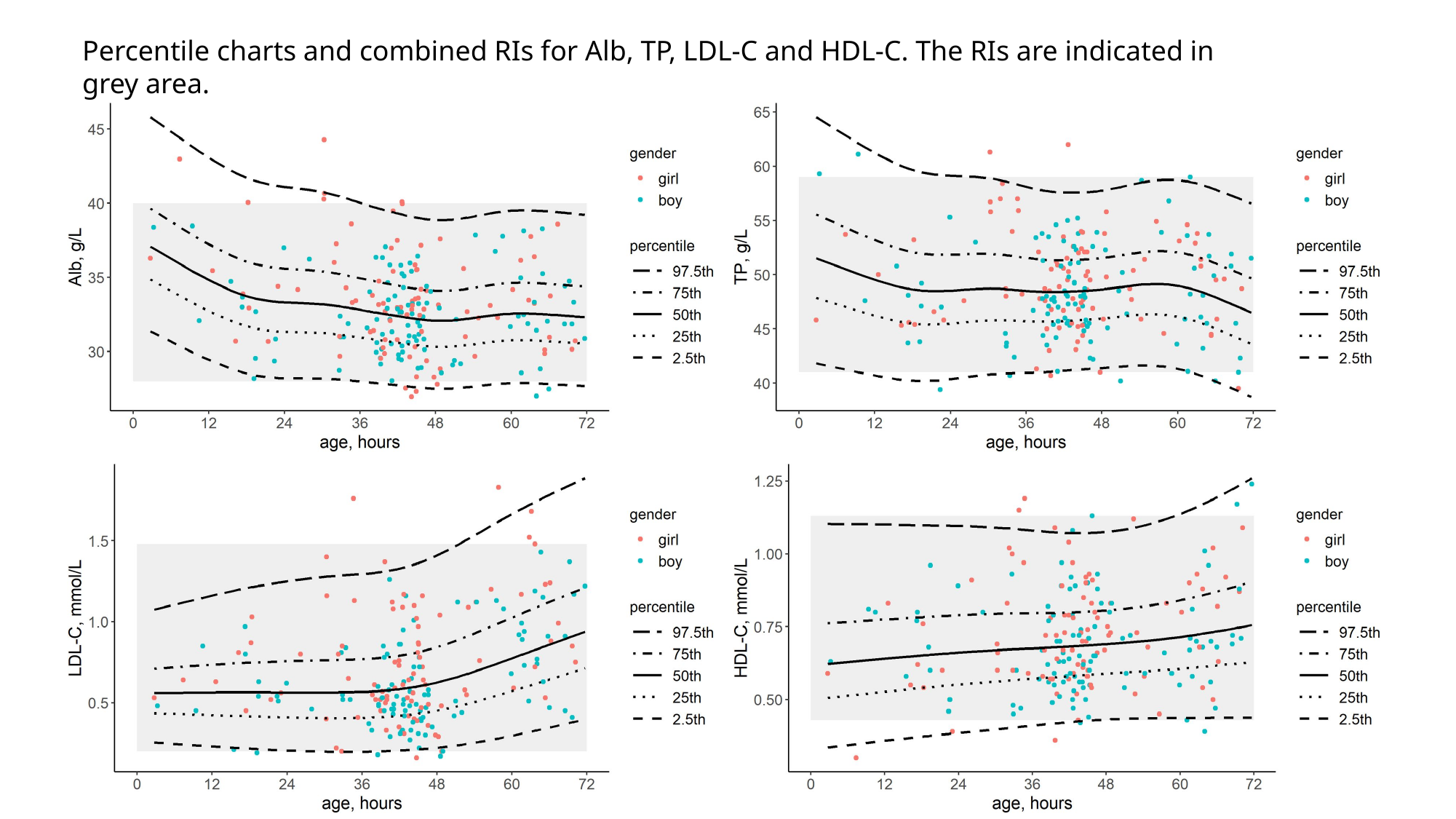

Percentile charts and combined RIs for Alb, TP, LDL-C and HDL-C. The RIs are indicated in grey area.
