# Supplementary material for: Reference intervals for 26 common biochemical analytes in term neonates in Jilin Province, China
Source: BMC Pediatr. 2021 Mar 31;21:156. doi: 10.1186/s12887-021-02565-8 (PMC8011145; doi:10.1186/s12887-021-02565-8)
Supplement: Supplementary file 3 — Additional file 3: Supplemental Table 2. Summary of reference intervals for 26 biochemistry analytes in comparison with other studies. [file 12887_2021_2565_MOESM3_ESM.docx]

**Supplemental Table 2**
Summary of reference intervals for 26 biochemistry analytes in comparison with other studies.

| **Analytes** | | **Current study [1]** | | | | **Manufacturer provided [2]** | | **Korea [3]** | **China [4]** | **China [5]** | | **Canada [6]** | |  |  |
| --- | --- | --- | --- | --- | --- | --- | --- | --- | --- | --- | --- | --- | --- | --- | --- |
|  |  | VITROS 5600 | | | | VITROS 5600 | | COBAS 8000 | HITACHI 7020 | OlYMPUS AU5400 | | VITROS 5600 | |  |  |
|  |  | Age | | | RI | RI for female | RI for male | RI | RI | Age | RI | Age | RI | |  |
| Chemistry | | | |  | |  |  |  |  |  |  |  |  | |  |
| CO_2_, mmol/L | 1-3 d | | | 16-28 | | 20-30 | |  | 11-27 |  |  |  |  | | |
| Cl, mmol/L | 1-3 d | | | 92-108 | | 98-107 | |  | 90-103 |  |  |  |  | | |
| K, mmol/L | 1-3 d | | | 3.3-5.4 | | 3.5-5.1 | |  | 3.64-6.00 |  |  |  |  | | |
| Na, mmol/L | 1-3 d | | | 131-140 | | 137-145 | |  | 130-142 |  |  |  |  | | |
| Ca, mmol/L | 1-3 d | | | 1.53-2.15 | | 2.10-2.55 | | 4.8-11.2 | 1.91-2.75 |  |  | 0-1 y | 1.98-2.9 | | |
| Mg, mmol/L | 1-3 d | | | 0.68-0.94 | | 0.7-1.0 | | 0.66-1.48 |  |  |  |  |  | | |
| P, mmol/L | 1-3 d | | | 1.70-2.75 | | 0.81-1.45 | | 0.13-1.19 |  |  |  | 0-14 d | 1.89-3.53 | | |
| Fe, μmol/L | 1 d | | | 5.2-14.6 | | 6.6-30.4 | 8.8-32.4 | 13.6-48.0 |  |  |  | 0-14 y | 4.8-25.3 | | |
|  | 2 d | | | 6.8-15.9 | |  |  |  |  |  |  |  |  | | |
|  | 3 d | | | 6.4-18.1 | |  |  |  |  |  |  |  |  | | |
| TIBC, μmol/L | 1-3 d | | | 32.9-52.4 | | 47.4-89.0 | 46.8-82.7 |  |  |  |  |  |  | | |
| BUN, mmol/L | 1 d | | | 0.71-5.72 | | 2.5-6.1 | 3.2-7.1 | 1.07-5.35 | 1.56-7.56 | 0-1 m | 0.8-5.1 |  |  | | |
|  | 2 d | | | 0.71-3.77 | |  |  |  |  |  |  |  |  | | |
|  | 3 d | | | 0.71-3.55 | |  |  |  |  |  |  |  |  | | |
| Cr, μmol/L | 1 d | | | 41-82 | | 46-92 | 58-110 | 18-80 | 16-110 | 0-3 d | 15.6-61.1 |  |  | | |
|  | 2 d | | | 38-76 | |  |  |  |  |  |  |  |  | | |
|  | 3 d | | | 31-65 | |  |  |  |  |  |  |  |  | | |
| UA, μmol/L | 1 d | | | 99-454 | | 149-369 | 208-506 |  | 82-446 | 0-1 m | 56.3-287.4 | 0-14 d | 168-751 | | |
|  | 2 d | | | 97-335 | |  |  |  |  |  |  |  |  | | |
|  | 3 d | | | 78-271 | |  |  |  |  |  |  |  |  | | |
| Enzymes | | | |  | |  |  |  |  |  |  |  |  | | |
| ALT, U/L | 1-3 d | | | 6-44 | | 9-52 | 21-72 | 3-20 | 1-32 | 0-15 d | 3-39 | 0-1 y | 17-69 | | |
| ALP, U/L | 1-3 d | | | 91-229 | | 38-126 | | 91-269 |  | 0-8 d | 43-344 | 0-14 d | 89-239 | | |
| AST, U/L | 1-3 d | | | 29-101 | | 14-36 | 17-59 | 16-73 | 22-168 | 0-8 d | 12-58 | 0-14 d | 29-213 | | |
| CHE, U/L | 1-3 d | | | 3299-6491 | | 4650-10440 | 5900-12220 |  |  | 0-1 m | 1.5-8.2 | 0-14 d | 3542-7370 | | |
| GGT, U/L | 1-3 d | | | 42-302 | | 12-43 | 15-73 | 29-502 |  | 0-3 m | 17-64 | 0-14 d | 19-196 | | |
| CK, U/L | 1-3 d | | | 95-715 | | 30-135 | 55-170 | 48-505 | 186-1074 |  |  |  |  | | |
| CKMB, U/L | 1-3 d | | | 12-40 | | - | |  | 14-94 |  |  |  |  | | |
| LDH, U/L | 1-3 d | | | 651-1577 | | 313-618 | | 200-926 | 429-1045 |  |  |  |  | | |
| Proteins | | | |  | |  |  |  |  |  |  |  |  | | |
| Alb, g/L | 1-3 d | | | 28-40 | | 35-50 | | 25-41 | 30.1-42.9 | 0-1 m | 21.6-46.3 | 0-14 d | 34-47 | | |
| TP, g/L | 1-3 d | | | 41-59 | | 63-82 | | 40-64 | 41.8-66.8 | 0-4 m | 42.2-65.7 | 0-14 d | 54-85 | | |
| Lipids/Lipoproteins | | | |  | |  |  |  |  |  |  |  |  | | |
| HDL-C, mmol/L | 1-3 d | | | 0.43-1.13 | | - | | 0.31-1.45 |  |  |  | 0-14 d | 0.38-1.03 | | |
| LDL-C, mmol/L | 1-3d | | | 0.20-1.48 | | - | | 0.1-1.53 |  |  |  |  |  | | |
| TG, mmol/L | 1-2 d | | | 0.38-1.25 | | - | | 0.11-0.89 |  |  |  | 0-14 d | 0.99-3.21 | | |
|  | 3 d | | | 0.32-1.84 | |  |  |  |  |  |  |  |  | | |
| TCHO, mmol/L | 1-2 d | | | 1.29-2.57 | | - | | <3.26 |  |  |  | 0-14 d | <1.29-3.20 | | |
|  | 3 d | | | 1.29-3.16 | |  |  |  |  |  |  |  |  | | |
| **Notes:** | | |  | | | | | | | | | | | | |
| Study type | | | [1,3,4] Prospective; [2]Not specified; [5] Retrospective; [6]Transference of RIs. | | | | | | | | | | | | |
| Age range studied | | | [1] 0-3 d; [2] Adult; [3] At birth; [4] 0-24 h; [5] 0-15 y; [6] 0-19 y. | | | | | | | | | | | | |
| Sample size | | | [1]195; [2] Not specified; [3] 79; [4] 400; [5] 63086; [6] Not available. | | | | | | | | | | | | |
| Gestational age | | | [1] 37-42; [2] Not available; [3] 26-42; [4] 37-42; [5,6] Not specified. | | | | | | | | | | | | |
| Sample type | | | [1,4] Arterial blood; [3] Umbilical cord; [2,5,6] Not specified. | | | | | | | | | | | | |
